# Replicon-Based Typing of IncI-Complex Plasmids, and Comparative Genomics Analysis of IncIγ/K1 Plasmids

**DOI:** 10.3389/fmicb.2019.00048

**Published:** 2019-01-29

**Authors:** Defu Zhang, Yuzong Zhao, Jiao Feng, Lingfei Hu, Xiaoyuan Jiang, Zhe Zhan, Huiying Yang, Wenhui Yang, Bo Gao, Jinglin Wang, Jianrong Li, Zhe Yin, Dongsheng Zhou

**Affiliations:** ^1^National and Local Joint Engineering Research Center of Storage, Processing and Safety Control Technology for Fresh Agricultural and Aquatic Products, Bohai University, Jinzhou, China; ^2^Fresh Food Storage and Processing Technology Research Institute of Liaoning Provincial Universities, Bohai University, Jinzhou, China; ^3^College of Food Science and Project Engineering, Bohai University, Jinzhou, China; ^4^State Key Laboratory of Pathogen and Biosecurity, Beijing Institute of Microbiology and Epidemiology, Beijing, China

**Keywords:** IncI-complex plasmids, IncIγ/K1, CTX-M, multi-drug resistance, plasmids

## Abstract

IncI-complex plasmids can be divided into seven subgroups IncI1, IncI2, IncIγ, IncB/O, IncK1, IncK2, and IncZ. In this study, a replicon-based scheme was proposed for typing IncI-complex plasmids into four separately clustering subgroups IncI2, IncI1/B/O, IncIγ/K1 and IncK2/Z, the last three of which were combined from IncI1 and IncB/O, IncIγ and IncK1, and IncK2 and IncZ, respectively. Four IncIγ/K1 plasmids p205880-NR2, p14E509-CTXM, p11011-CTXM and p61806-CTXM were fully sequenced and compared with IncIγ/K1 reference pCT, IncI2 reference R721, IncI1/B/O reference R64 and IncK2/Z reference pO26-CRL-125. These plasmids shared conserved gene organization in the replication and conjugal transfer regions, but displaying considerable sequence diversity among different subgroups. Remarkable modular differences were observed in the maintenance and transfer leading regions. p205880-NR2 contained no resistance genes or accessory modules, while the other seven plasmids acquired one or more accessory modules, which harbored mobile elements [including unit transposons, insertion sequence (IS)-based transposition units and individual IS elements] and associated resistance markers (especially including those involved in resistance to β-lactams, aminoglycosides, tetracyclins, phenicols, streptomycins, trimethoprims, sulphonamides, tunicamycins and erythromycins). Data presented here provided a deeper insight into diversification and evolution of IncI-complex plasmids.

## Introduction

Plasmids of the I incompatibility complex (IncI-complex) produce I-type pili. Based on morphological and serological similarities of their pili, IncI-complex plasmids can be divided into at least seven subgroups, namely IncI1 ( = IncIα or Com1), IncI2 ( = IncIδ), IncIγ, IncB/O, IncK1, IncK2, and IncZ (Tschäpe and Tietze, 2010; Papagiannitsis et al., 2011; Rozwandowicz et al., 2017). IncI-complex plasmids are low copy-number, narrow-host-range, conjugative plasmids, varying in size from 50 to 250 kb. The backbone of an IncI-complex plasmid can be dividedinto regions of replication, maintenance, transfer leading and conjugal transfer (Sampei et al., 2010). Two protein genes (*repZ* and *repY*), one regulatory RNA gene (*inc*) and four functional DNA sequences (*CIS*, *oriV*, *ter*, and iterons) in the replication region are essential for plasmid replication and copy number control (Sampei et al., 2010). The partitioning genes *parA* and *parB* in the maintenance region are responsible for active partition of replicated DNA into daughter cells during cell division. Two gene clusters *tra* and *pil* in the conjugal transfer region encoded the thick rigid pilus as the primary pili implicated in conjugation transfer, and the thin flexible pilus increasing conjugation rate in liquid medium, respectively (Bradley, 1984). A multiple inversion system named shufflon mediates rearrangement of PilV protein by a plasmid-encoded site-specific recombinase Rci (Komano et al., 1987). This recombination event selects one of seven different *pilV* genes, which is responsible for the determination of recipient specificity (Komano et al., 1994).

The purpose of this study is to provide a deeper insight into genomic diversity and evolution of IncI-complex plasmids. First, a replicon-based scheme was established to divide IncI-complex plasmids into four subgroups IncI2, IncI1/B/O, IncIγ/K1, and IncK2/Z. Then, four IncIγ/K1 plasmids p14E509-CTXM, p11011-CTXM, p61806-CTXM, and p205880-NR2 were fully sequenced and compared to the reference plasmids pCT (accession number FN868832) (Cottell et al., 2011), R721 (accession number AP002527), R64 (accession number AP005147) (Sampei et al., 2010), and pO26-CRL-125 (accession number KC340959) (Venturini et al., 2013) of the above four subgroups.

## Materials and Methods

### Bacterial Isolates

*Escherichia coli* 14E509 was isolated in 2014 from a urine specimen of an infant with pneumonia in a public hospital in Henan City, China. *E. coli* 11011 was isolated in 2014 from a bile specimen of an elderly patient with bile duct calculus in a public hospital in Ningbo City, China. *E. coli* 61806 was isolated in 2015 from a whole blood specimen of an elderly patient with septic shock in a teaching hospital in Henan City, China. *Klebsiella pneumoniae* 205880 was isolated in 2012 from a sputum specimen of an elderly patient with pneumonia in a teaching hospital in Chongqing City, China.

### Genomic DNA Sequencing and Sequence Assemble

Genomic DNA was isolated from each isolate using a Qiagen Blood & Cell Culture DNA Maxi Kit (Qiagen, Hilden, Germany). The genomic DNA of strain 61806 was sequenced from a mate-pair libraries with average insert size of 5 kb (ranged from 2 to 10 kb) using a MiSeq sequencer (Illumina, CA, United States). Quality control, removing adapters and low quality reads, were performed using *Trimmomatic* 0.36 (Bolger et al., 2014). The filtered clean reads were then assembled using *Newbler* 2.6 (Nederbragt, 2014), followed by extraction of the consensus sequence with *CLC Genomics Workbench* 3.0 (Qiagen Bioinformatics). Gaps between contigs were filled using a combination of PCR and Sanger sequencing using an ABI 3730 Sequencer (LifeTechnologies, CA, United States), and *Gapfiller* V1.11 (Boetzer and Pirovano, 2012) was used for gap closure.

For all the other three isolates (14E509, 11011 and 205880), genome sequencing was performed with a sheared DNA library with average size of 15 kb (ranged from 10 to 20 kb) on a PacBio RSII sequencer (Pacific Biosciences, CA, United States), as well as a paired-end library with an average insert size of 400 bp (ranged from 150 to 600 kb) on a HiSeq sequencer (Illumina, CA, United States). The paired-end short Illumina reads were used to correct the long PacBio reads utilizing *proovread* (Hackl et al., 2014), and then the corrected PacBio reads were assembled *de novo* utilizing *SMARTdenovo* (available from https://github.com/ruanjue/smartdenovo).

### Sequence Annotation and Genome Comparison

Open reading frames and pseudogenes were predicted using *RAST* 2.0 (Brettin et al., 2015) combined with *BLASTP/BLASTN* (Boratyn et al., 2013) searches against the *UniProtKB/Swiss-Prot* database (Boutet et al., 2016) and the *RefSeq* database (O’Leary et al., 2016). Annotation of resistance genes, mobile elements, and other features was carried out using the online databases including *CARD* (Jia et al., 2017), *ResFinder* (Zankari et al., 2012), *ISfinder* (Siguier et al., 2006), *INTEGRALL* (Moura et al., 2009), and *Tn Number Registry* (Roberts et al., 2008). Multiple and pairwise sequence comparisons were performed using *MUSCLE* 3.8.31 (Edgar, 2004) and *BLASTN*, respectively. Gene organization diagrams were drawn in *Inkscape* 0.48.1^1^.

### Phylogenetic Analysis

The nucleotide sequences of *repZ* coding regions of indicative plasmids were aligned using *MUSCLE* 3.8.31 (Edgar, 2004). Unrooted neighbor-joining trees were generated from aligned *repZ* sequences using *MEGA7* (Kumar et al., 2016), and evolutionary distances were estimated using maximum composite likelihood method, with a bootstrap iteration of 1000.

### Plasmid Conjugal Transfer

Plasmid conjugal transfer experiments were carried out with the rifampin-resistant *E*. *coli* EC600 or the sodium azide-resistant *E. coli* J53 being used as a recipient and each of the 14E509, 11011 and 61806 isolates as a donor. Three milliliters of overnight cultures of each of donor and recipient bacteria were mixed together, harvested and resuspended in 80 μl of Brain Heart Infusion (BHI) broth (BD Biosciences, NJ, United States). The mixture was spotted on a 1 cm^2^ hydrophilic nylon membrane filter with a 0.45 μm pore size (Millipore, MA, United States) that was placed on BHI agar (BD Biosciences, NJ, United States) plate and then incubated for mating at 37°C for 12 to 18 h. Bacteria were washed from filter membrane and spotted on Muller–Hinton (MH) agar (BD Biosciences, NJ, United States) plates containing 1000 μg/ml rifampin or 200 μg/ml sodium azide together with 200 μg/ml ampicillin for selecting an *E. coli* transconjugant that carrying *bla*_CTX-M_.

### Double-Disk Synergy Test

To detect ESBL activity, the double-disk synergy test was performed as recommended by the National Committee for Clinical and Laboratory Standards Institute (CLSI) guideline (CLSI, 2015). Briefly, the bacterial strains tested were spread onto MH agar plates and four disks containing cefotaxime (30 μg), ceftazidime (30 μg), cefotaxime (30 μg) plus clavulanic acid (10 μg), and ceftazidime (30 μg) plus clavulanic acid (10 μg) were applied to each agar plate. The agar plates were incubated overnight at 37°C, and the production of ESBL was inferred when the zone of either cephalosporin was ≥5 mm larger than those without clavulanic acid.

### Bacterial Antimicrobial Susceptibility Test

Bacterial antimicrobial susceptibility was tested by BioMérieux VITEK 2 and interpreted as per the Clinical and Laboratory Standards Institute (CLSI) guidelines (CLSI, 2015).

### Nucleotide Sequence Accession Numbers

The complete sequence of plasmids p14E509-CTXM, p11011-CTXM, p205880-NR2, and p61806-CTXM were submitted to GenBank under accession numbers MG764547, MF344575, MF344577, and MF344576, respectively.

## Results and Discussion

### Replicon-Based Typing of IncI-Complex Plasmids

**FIGURE 1:**
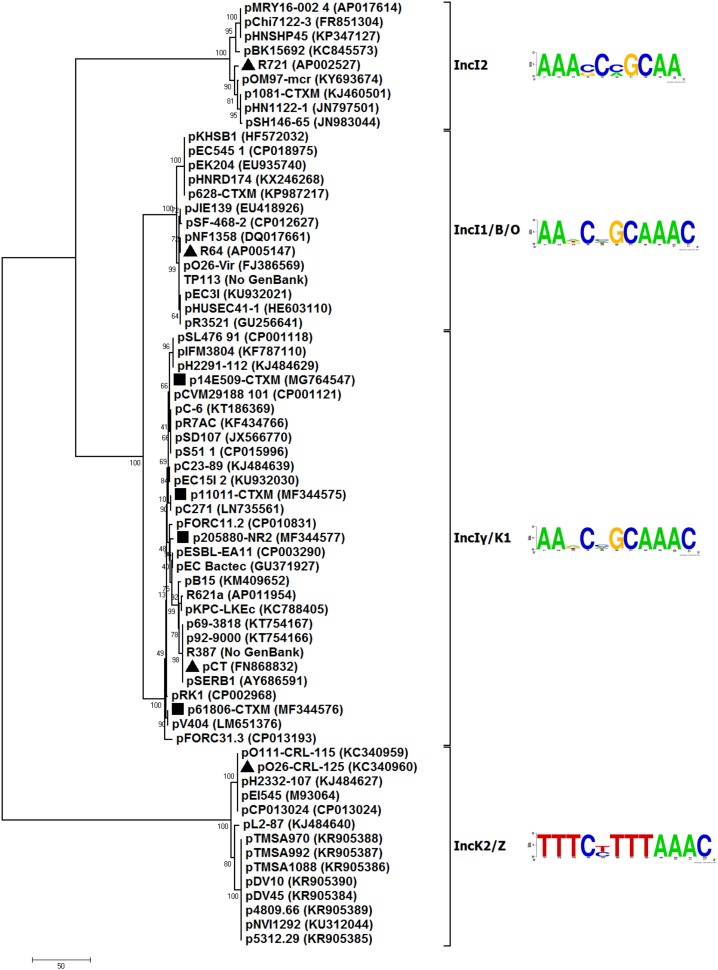
Neighbor-joining phylogenetic tree. The degree of support (percentage) for each cluster of associated taxa, as determined by bootstrap analysis, is shown next to each branch. The bar corresponds to the scale of sequence divergence. Triangles indicate reference plasmids for IncIγ/K1, IncI2, IncI1/B/O, and IncK2/Z subgroups, while squares denote four plasmids sequences in this study.

To better understand the evolutionary relationship of IncI-complex plasmids, a phylogenetic tree (Figure 1) was constructed from the nucleotide sequences of *repZ* (replication initiation) coding regions of 66 representative (arbitrarily selected) sequenced IncI-complex plasmids (Supplementary Table S1). These 66 plasmids could be divided into four separately clustering subgroups, namely IncI2, IncI1/B/O, IncIγ/K1, and IncK2/Z, the last three of which were combined from IncI1 and IncB/O, IncIγ and IncK1, and IncK2 and IncZ, respectively. As shown by pairwise comparison of *repZ* sequences, plasmids within each of these four subgroups shared >95% nucleotide identity, by contrast <95% sequence identity was observed between different subgroups (Supplementary Table S2).

Putative iterons (RepZ-binding sites) were found to be located 176 to 301 bp downstream of *repZ* for all these 66 plasmids analyzed (Supplementary Table S1). Plasmids within each of these four subgroups shared a conserved iteron motif (Figure 1) and an identical iteron copy number (Supplementary Table S1). Iteron motifs from different subgroups dramatically differed from one another except for IncIγ/K1 and IncI1/B/O (these two had very similar iteron motifs). IncK2/Z plasmids had two copy numbers of iteron, while plasmids of all the other subgroups had three copy numbers.

Plasmid compatibility among original seven subgroups of IncI-complex had been partially validated experimentally (Praszkier et al., 1991; Rozwandowicz et al., 2017). IncIγ reference plasmid R621a was compatible with IncI1 plasmids (Bird and Pittard, 1982), and IncK2 plasmids was compatible with IncK1 reference plasmid pCT and IncB/O reference plasmid pR3521 (Rozwandowicz et al., 2017). IncZ plasmids were compatible with IncK1, IncIγ and IncI1 plasmids (Praszkier and Pittard, 2005). Plasmid compatibility among the four IncI-complex subgroups IncIγ/K1, IncI1/B/O, IncK2/Z, and IncI2 needed to be elucidated.

### Overview of Sequenced IncIγ/K1 Plasmids

The four IncIγ/K1 plasmids p14E509-CTXM, p11011-CTXM, p61806-CTXM, and p205880-NR2 sequenced in the present work varied in size from about 83 kb to nearly 132 kb with variation in corresponding number of predicted ORFs (Table 1). The former three plasmids integrated various accessory modules (defined as acquired DNA regions associated with or bordered by mobile elements), which harbored resistance genes and metabolic gene clusters, and associated unit transposons, insertion sequence (IS)-based transposition units and individual IS elements. By contrast, p205880-NR2 contained no resistance genes or accessory modules (Table 1 and Supplementary Figure S1), thereby representing a prototype IncIγ/K1 plasmid.

**Table 1:** Major features of plasmids analyzed.

Category	IncI1/B/O plasmid	IncIγ/K1 plasmids					IncK2/Z plasmid	IncI2 plasmid
				
	R64^@^	p205880-NR2^$^	p14E509-CTXM^$^	p11011-CTXM^$^	p61806-CTXM^$^	pCT^@^	pO26-CRL-125^@^	R721^@^
Total length (bp)	120,826	82,822	112,544	131,779	104,270	93,629	124,908	75,582
Total number of ORFs	142	100	128	159	138	119	141	109
Mean G+C content, %	49.6%	50.3%	50.1%	50.9%	52.5%	52.7%	53.3%	42.6%
Length of backbone sequence (bp)	103,582	82,822	99,279	97,310	94,485	83,915	96,005	60,062
Accessory modules	IS*2*:Tn*5393l* region^#^	Not found	*bla*_CTX-M-14_-containing region^#^	*bla*_CTX-M-65_-containing region^#^	*bla*_CTX-M-14_-containing region^#^, and ΔIS*26*	*bla*_CTX-M-14_-containing region^#^, IS*Kox3*, IS*Cro1*, and IS*Sso4*	Tn*6414*-ΔTn*1721* region^#^	Tn*7*^#^, and IS*150*
	Do not carrying *bla*_CTX-M_ genes		Carrying *bla*_CTX-M-14or65_ genes				Do not carrying *bla*_CTX-M_ genes	


*^$^, sequenced in this study; ^@^, reference plasmids derived from GenBank; ^#^, carrying resistance genes. The sequence of each plasmid was divided into one or more accessory modules (defined as acquired DNA regions associated with or bordered by mobile elements) and the remaining backbone regions.*

**FIGURE 2:**
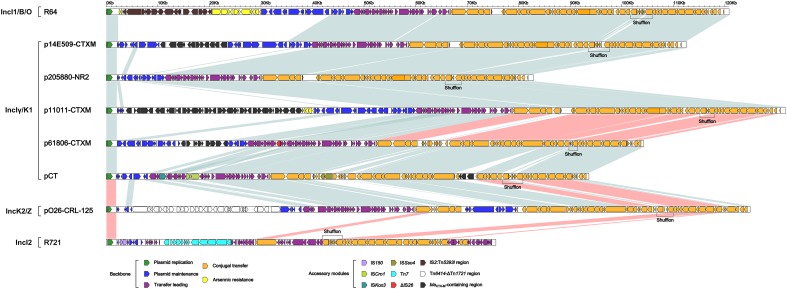
Linear comparison of sequenced plasmids. Genes are denoted by arrows and colored based on gene function classification. Shading regions denote regions of homology (light blue: >95% nucleotide identity; light red: very low nucleotide identity with conserved gene functions).

These four IncIγ/K1 plasmids, together with pCT (IncIγ/K1 reference), R721 (IncI2 reference), R64 (IncI1/B/O reference) and pO26-CRL-125 (IncK2/Z reference), were included in a genomic comparison. Considerable modular and sequence diversity were found among the backbones of these eight plasmids (Supplementary Table S3 and Figure 2). IncIγ/K1 plasmids p61806-CTXM and pCT shared >99% nucleotide identity across >81% of their backbone sequences. IncIγ/K1 plasmids p14E509-CTXM, p11011-CTXM and p205880-NR2, and IncI1/B/O plasmid R64 shared >99% nucleotide identity across >74% of their backbone sequences. pCT and IncK2/Z plasmid pO26-CRL-125 shared >95% nucleotide identity over >70% of their backbone sequences. The backbone of IncI2 plasmid R721 displayed very low level (<3% BLAST coverage and >82% nucleotide identity) of sequence identity to the other seven plasmids.

Each of these eight plasmids had its unique backbone genes or gene loci, especially including those in the maintenance regions. All these plasmids shared the backbone genes or gene loci *inc-repY-repZ-CIS-oriV*-ter (plasmid replication), *parA* (maintenance), and *nicA*, *rlx*, *rci*, *pil* and *tra* (conjugal transfer) and also the conserved gene organization in the replication and conjugal transfer regions, but with remarkable nucleotide and amino acid diversity among different subgroups.

Notably, p61806-CTXM and pCT had IncIγ/K1-type conjugal transfer regions, while p14E509-CTXM, p11011-CTXM and p205880-NR2 had IncI1/B/O-type conjugal transfer regions as observed in R64. It was speculated that homologous recombination-mediated horizontal transfer of conjugal transfer regions occurred between IncIγ/K1 and IncI1/B/O.

### Accessory Modules

p14E509-CTXM, p11011-CTXM, p61806-CTXM, pCT, R721, R64 and pO26-CRL-125 carried different profiles of accessory modules (Table 1). R721 harbored two separate accessory modules, namely Tn*7* (see reference Zhan et al., 2018 for gene organization) and IS*150*. R64 contained a single 17.2 kb accessory module, designated the IS*2*:Tn*5393l* region, which was generated from insertion of Tn*5393l* [a novel derivative of prototype Tn*5393c* (Cain and Hall, 2011)] into IS*2* (Supplementary Figure S2). pO26-CRL-125 contained a 28.9 kb Tn*6414*-ΔTn*1721* region (as its sole accessory module) with a Tn*6414-oriV*_IncP-1α_–ΔTn*1721* structure (Supplementary Figure S3). Tn*6414* was a novel derivative of Tn*21*, which was resulted from insertion of In2 into a backbone structure carrying the core transposition module *tnpAR-res-tnpM* together with the *mer* locus. Tn*6414* differed from Tn*21* by insertion of In13 instead of In2. In13 is atypical due to integration of two overlapping transposons Tn*6029* and Tn*4325* downstream of the *dfrA*5 single-gene cassette.

**FIGURE 3:**
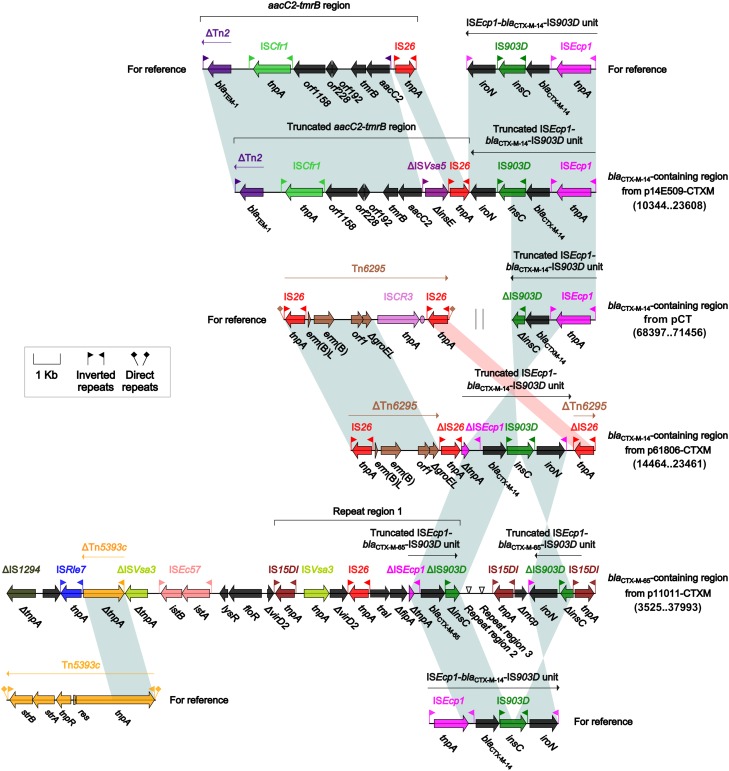
*bla*_CTX-M_-containingregions. Genes are denoted by arrows. Genes, mobile elements and other features are colored based on function classification. Shading denotes regions of homology (>95% nucleotide identity). Numbers in brackets indicate nucleotide positions within corresponding plasmids. The accession numbers of the *aacC2–tmrB* region, the *ISEcp1–bla*_CTX-M-14_*–*IS*903D* unit, Tn*6295* and Tn*5393c* for reference are JX101693, KP987215, KX646543, and AF262622, respectively.

IncI-complex plasmids carry a wide range of resistance genes (Supplementary Table S4), among which extended-spectrum β-lactamase (ESBL) genes are often identified. Indeed, each of the four IncIγ/K1 plasmids p14E509-CTXM, pCT, p61806-CTXM, and p11011-CTXM carried a *bla*_CTX-M_-containing region (Figure 3); besides, individual IS elements were found as additional accessory modules: IS*Kox3*, IS*Cro1*, and IS*Sso4* in pCT, and ΔIS*26* in p61806-CTXM. In these four *bla*_CTX-M_-containing regions, different truncated versions of prototype IS*Ecp1-bla*_CTX-M-14 or 65_-IS*903D* unit were connected with additional resistance regions: truncated *aacC2-tmrB* region in p14E509-CTXM, ΔTn*6295* in p61806-CTXM, and the other three copies of truncated IS*Ecp1-bla*_CTX-M-14 or 65_-IS*903D* unit in p11011-CTXM. Complex homologous recombination, which was probably mediated by the common region IS*15DI*, would be involved in assembly of these repeated *bla*_CTX-M_-containing regions. Connection of *bla*_CTX-M_-containing regions with additional resistance regions led to MDR of p14E509-CTXM and p11011-CTXM.

### Transferability and Antimicrobial Susceptibility

The *bla*_CTX-M_-carrying plasmids p14E509-CTXM, p11011-CTXM and p61806-CTXM could be transferred from the wild-type isolates into *E. coli* J53 or EC600 through conjugation, generating three transconjugants 14E509-CTXM-J53, 11011-CTXM-EC600 and 61806-CTXM-EC600, respectively. All these three transconjugants had the ESBL activity (data not shown) and, as expected, were resistant to ampicillin, piperacillin, cefazolin, cefuroxime and ceftazidime but remained susceptible to ampicillin/sulbactam, piperacillin/tazobactam, imipenem and meropenem (Supplementary Table S5).

## Ethics Statement

The use of human specimens and all related experimental protocols were approved by the Committee on Human Research of the Henan Provincial People’s Hospital, Ningbo Medical Treatment Center Lihuili Hospital, the First Affiliated Hospital of Henan University, and the First Affiliated Hospital of Chongqing Medical University, and carried out in accordance with the approved guidelines. The research involving biohazards and all related procedures were approved by the Biosafety Committee of the Beijing Institute of Microbiology and Epidemiology.

## Author Contributions

DsZ and ZY conceived the study and designed experimental procedures. DfZ, YZ, JF, LH, and XJ performed the experiments. ZZ, HY, WY, BG, JW, and JL analyzed the data. ZZ, YZ, and JF contributed reagents and materials. DsZ, ZY, DfZ and YZ wrote this manuscript.

## Conflict of Interest Statement

The authors declare that the research was conducted in the absence of any commercial or financial relationships that could be construed as a potential conflict of interest.

## Footnotes

1 https://inkscape.org/en/

## Supplementary Material

Figure S1 Plasmid schematic maps. Genes are denoted by arrows, and the backbone and accessory module regions are highlighted in black and gray, respectively. The innermost circle presents GC-skew [(G-C)/(G+C)], with a window size of 500 bp and a step size of 20 bp. The next-to-innermost circle presents GC content.

Figure S2 The IS*2*:Tn*5393l* region from R64. Genes are denoted by arrows. Genes, mobile elements and other features are colored based on function classification.

Figure S3 The MDR region from pO26-CRL-125. Genes are denoted by arrows. Genes, mobile elements and other features are colored based on function classification. Shading denotes regions of homology (>95% nucleotide identity). Numbers in brackets indicate nucleotide position within pO26-CRL-125. The accession numbers of Tn*1721* and Tn*21* for reference are X61367 and AF071413, respectively.
