# Supplementary figures and images for: Replicon-Based Typing of IncI-Complex Plasmids, and Comparative Genomics Analysis of IncIγ/K1 Plasmids
Source: Front Microbiol. 2019 Jan 29;10:48. doi: 10.3389/fmicb.2019.00048 (PMC6361801; doi:10.3389/fmicb.2019.00048)

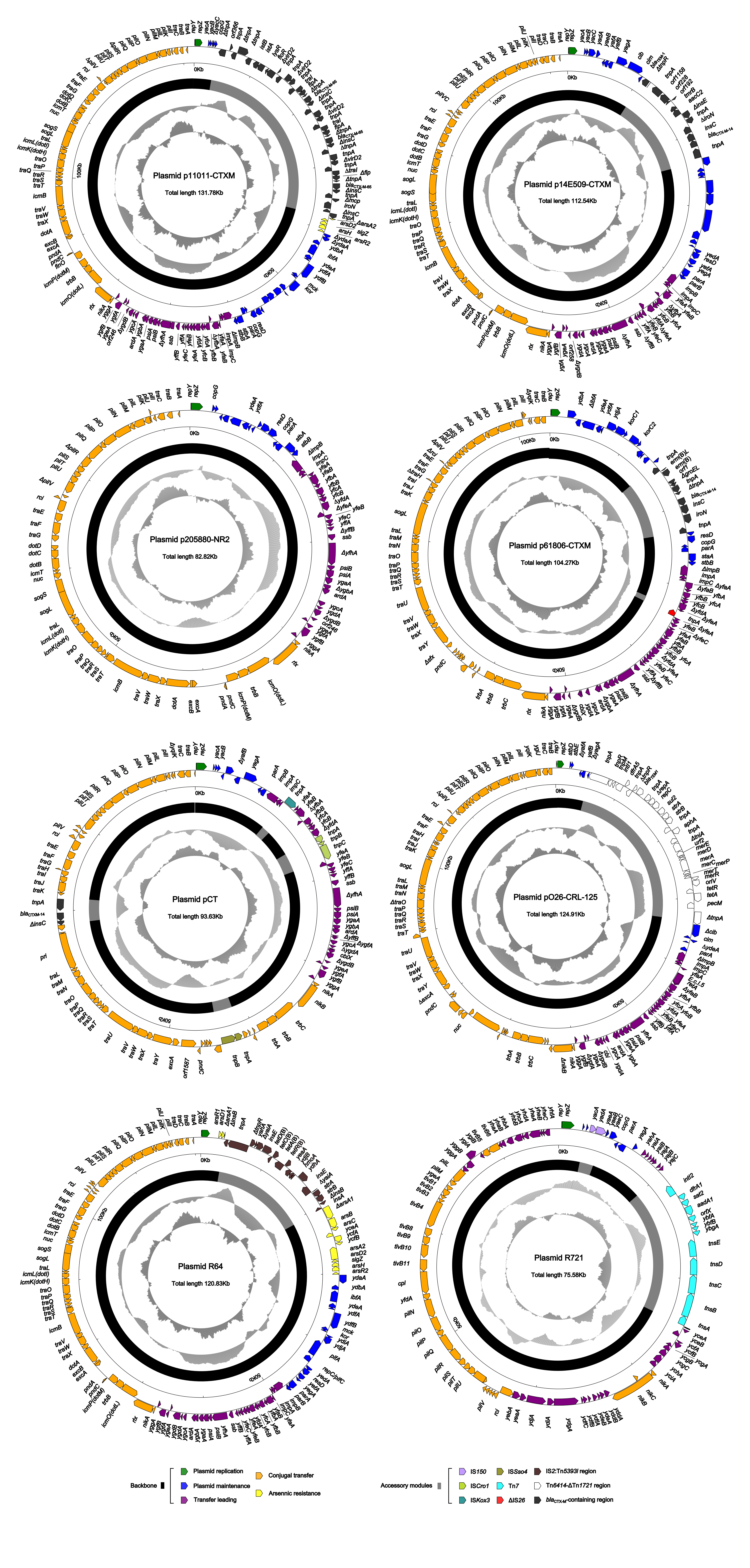

Supplement: Figure S1 — Plasmid schematic maps. Genes are denoted by arrows, and the backbone and accessory module regions are highlighted in black and gray, respectively. The innermost circle presents GC-skew [(G-C)/(G+C)], with a window size of 500 bp and a step size of 20 bp. The next-to-innermost circle presents GC content. [file Image_1.TIF]

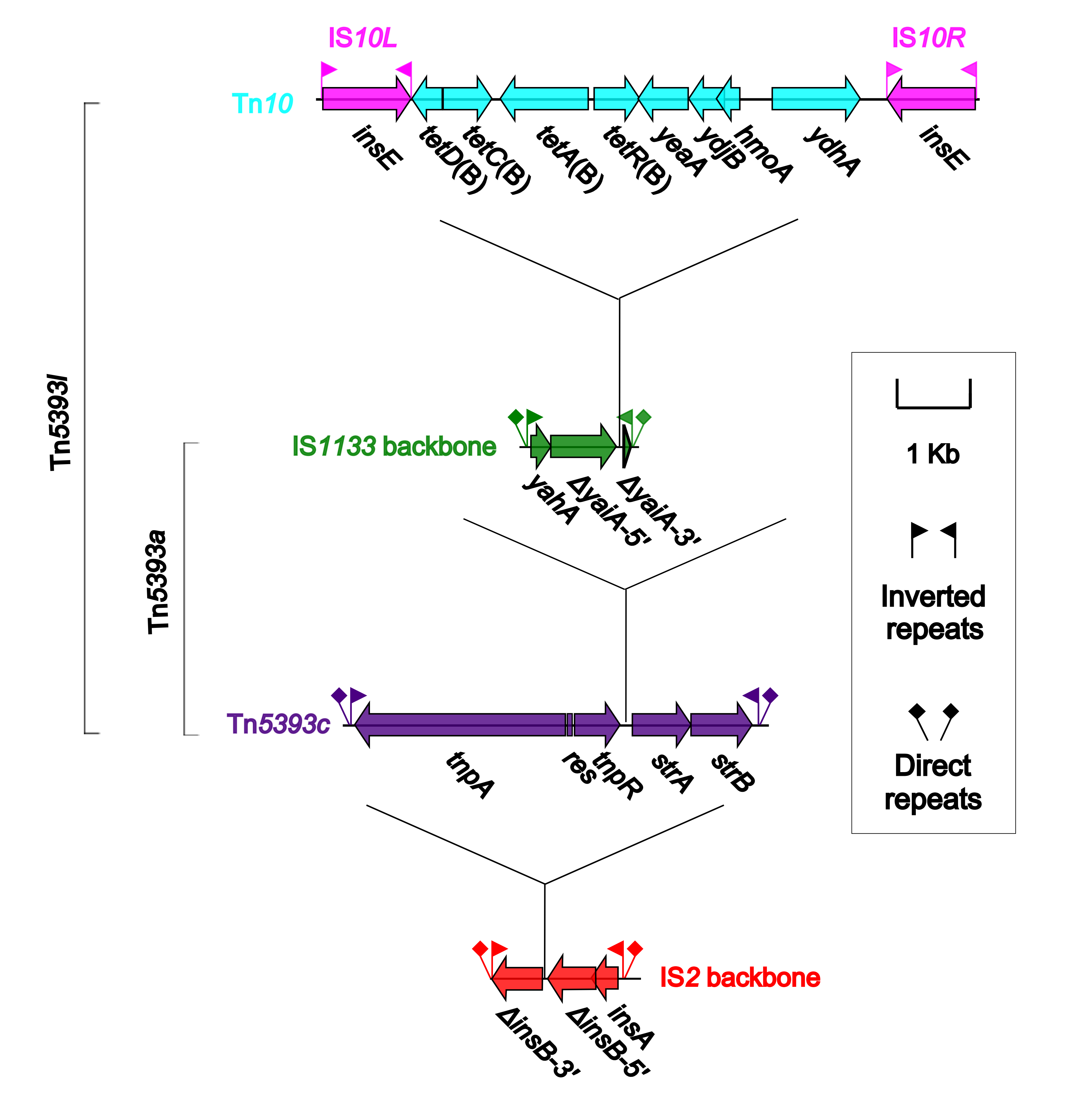

Supplement: Figure S2 — The IS2:Tn5393l region from R64. Genes are denoted by arrows. Genes, mobile elements and other features are colored based on function classification. [file Image_2.TIF]

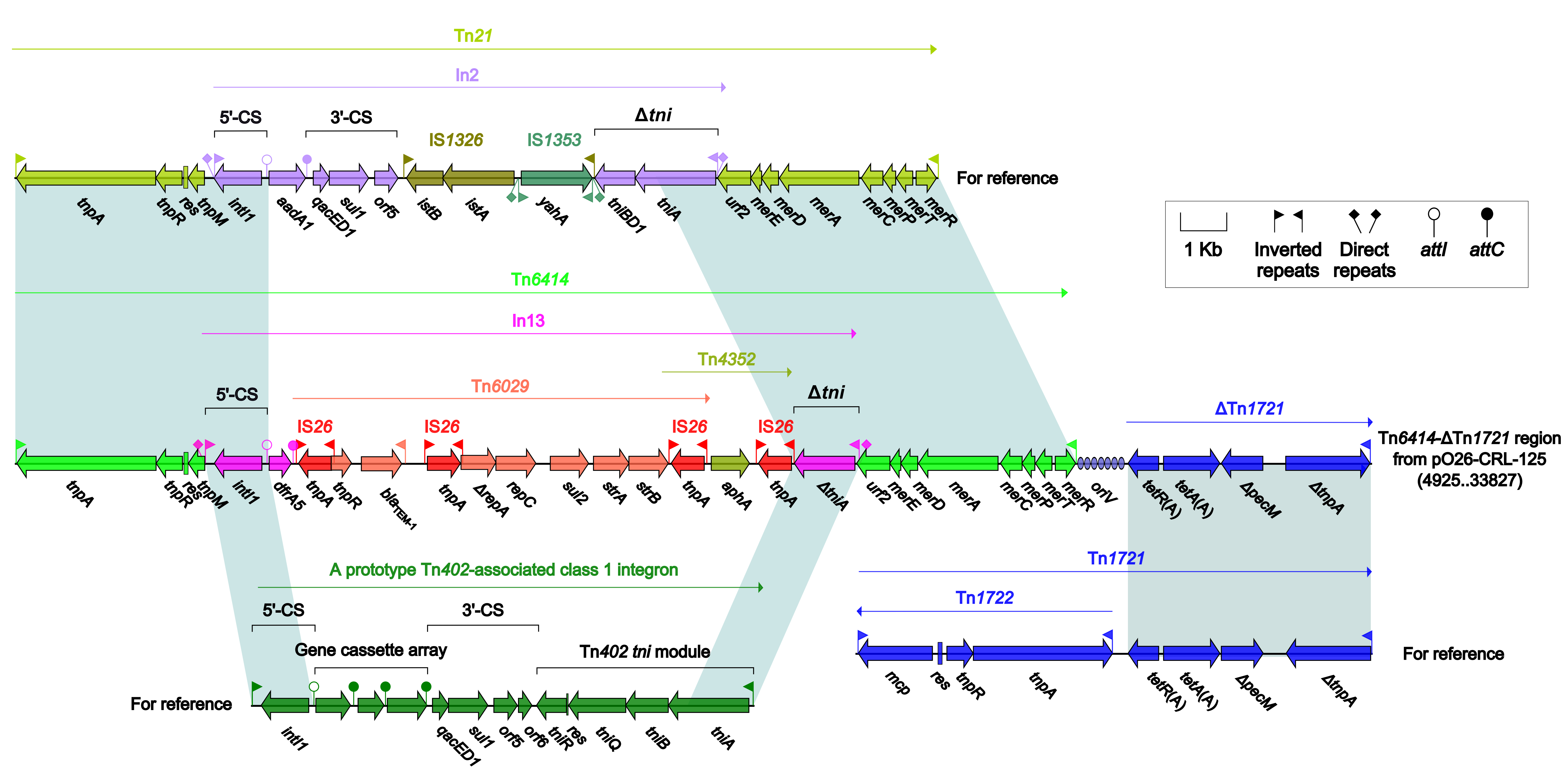

Supplement: Figure S3 — The MDR region from pO26-CRL-125. Genes are denoted by arrows. Genes, mobile elements and other features are colored based on function classification. Shading denotes regions of homology (>95% nucleotide identity). Numbers in brackets indicate nucleotide position within pO26-CRL-125. The accession numbers of Tn1721 and Tn21 for reference are X61367 and AF071413, respectively. [file Image_3.TIF]
