# Supplementary material for: Replicon-Based Typing of IncI-Complex Plasmids, and Comparative Genomics Analysis of IncIγ/K1 Plasmids
Source: Front Microbiol. 2019 Jan 29;10:48. doi: 10.3389/fmicb.2019.00048 (PMC6361801; doi:10.3389/fmicb.2019.00048)
Supplement: Supplementary file 7 [file Table_4.docx]

**TABLE S4 | Antimicrobial drug susceptibility profiles**

| **Antibiotics** | **MIC (mg/L) /antimicrobial susceptibility** | | | | | | | | |
| --- | --- | --- | --- | --- | --- | --- | --- | --- | --- |
|  | **11011** | **11011-**  **CTXM-**  **EC600** | **61806** | **61806-**  **CTXM-**  **EC600** | **14E509** | **14E509-**  **CTXM-**  **J53** | **205880** | **EC600** | **J53** |
| Ampicillin | >32/R | >32/R | >32/R | >32/R | >32/R | >32/R | >32/R | 16/R | 8/S |
| Ampicillin/  Sulbactam | >32/R | >32/R | >32/R | >32/R | >32/R | >32/R | >32/R | 4/S | ≤2/S |
| Piperacillin | ≥128/R | ≥128/R | ≥128/R | ≥128/R | ≥128/R | ≥128/R | ≥128/R | ≤4/S | ≤4/S |
| Piperacillin/  Tazobactam | ≥128/R | ≤4/S | ≥128/R | ≤4/S | ≥128/R | ≤4/S | ≥128/R | ≤4/S | ≤4/S |
| Cefazolin | ≥64/R | ≥64/R | ≥64/R | ≥64/R | ≥64/R | ≥64/R | ≥64/R | ≤4/S | ≤4/S |
| Cefuroxime | ≥64/R | ≥64/R | ≥64/R | ≥64/R | ≥64/R | ≥64/R | ≥64/R | 4/S | 4/S |
| Ceftazidime | ≥64/R | 4/S | ≥64/R | 16/R | ≥64/R | ≤1/S | ≥64/R | ≤4/S | ≤4/S |
| Imipenem | ≥16/R | ≤1/S | ≥16/R | ≤1/S | ≥16/R | ≤1/S | ≥16/R | ≤1/S | ≤1/S |
| Meropenem | 8/R | ≤0.25/S | ≥16/R | ≤0.25/S | 8/R | ≤0.25/S | 8/R | ≤0.25/S | ≤0.25/S |
| Amikacin | ≥64/R | ≤2/S | ≥64/R | ≥64/R | ≥64/R | ≤2/S | ≥64/R | ≤2/S | ≤2/S |
| Tobramycin | ≥16/R | ≤1/S | ≥16/R | ≥16/R | ≥16/R | 8/I | ≥16/R | ≤1/S | ≤1/S |
| Ciprofloxacin | ≥4/R | ≤0.25/S | ≥4/R | ≤0.25/S | ≥4/R | 1/S | ≥4/R | ≤0.25/S | ≤0.25/S |
| Levofloxacin | ≥8/R | ≤0.5/S | ≥8/R | ≤0.5/S | ≥8/R | 1/S | ≥8/R | ≤0.25/S | ≤0.25/S |
| Trimethoprim/  Sulfamethoxazole | ≥320/R | ≤20/S | ≥320/R | ≤20/S | ≥320/R | ≤20/S | ≥320/R | ≤20/S | ≤20/S |

S=sensitive; R=resistant; I=intermediately resistant
