# Supplementary material for: Replicon-Based Typing of IncI-Complex Plasmids, and Comparative Genomics Analysis of IncIγ/K1 Plasmids
Source: Front Microbiol. 2019 Jan 29;10:48. doi: 10.3389/fmicb.2019.00048 (PMC6361801; doi:10.3389/fmicb.2019.00048)
Supplement: Supplementary file 8 [file Table_5.docx]

**TABLE S5 | Resistance genes in plasmids analyzed**

| **Plasmids** | **Resistance marker** | **Resistance phenotype** | **Nucleotide position** | **Region located** |
| --- | --- | --- | --- | --- |
| R64 | *ars* | Arsenic resistance | 2470..3306  20545..24018  26118..30022 | Backbone |
|  | *tet* | Tetracycline resistance | 10009..13129 | IS*2*:Tn*5393l* region |
|  | *strAB* | Streptomycin resistance | 18010..19649 |  |
| p14E509-CTXM | *bla*_TEM-1_ | β-lactam resistance | 10491..11351 | *bla*_CTX-M-14_-containing region |
|  | *tmrB* | Tunicamycin resistance | 15768..16310 |  |
|  | *aacC2* | Aminoglycoside resistance | 16323..17183 |  |
|  | *bla*_CTX-M-14_ | β-lactam resistance | 21035..21895 |  |
| p11011-CTXM | *floR* | Phenicol resistance | 12002..13216 | *bla*_CTX-M-65_-containing region |
|  | *bla*_CTX-M-65_ | β-lactam resistance | 19090..19965  25933..26808  32769..33644 |  |
|  | *ars* | Arsenic resistance | 37989..40321 | Backbone |
| p61806-CTXM | *erm*(B) | Erythromycin resistance | 15576..16313 | *bla*_CTX-M-14_-containing region |
|  | *bla*_CTX-M-14_ | β-lactam resistance | 19331..20182 |  |
| pCT | *bla*_CTX-M-14_ | β-lactam resistance | 68883..69743 | *bla*_CTX-M-14_-containing region |
| pO26-CRL-125 | *dfrA5* | Trimethoprim resistance | 10335..10808 | Tn*6414*-ΔTn*1721* region |
|  | *bla*_TEM-1_ | β-lactam resistance | 12249..13109 |  |
|  | *sul2* | Sulphonamide resistance | 16326..17141 |  |
|  | *strAB* | Streptomycin resistance | 17246..18840 |  |
|  | *aphA* | Aminoglycoside resistance | 19756..20571 |  |
|  | *mer* | Mercury resistance | 23559..27521 |  |
|  | *tet* | Tetracycline resistance | 28638..30593 |  |
| R721 | *dfrA2* | Trimethoprim resistance | 12484..12957 | Tn*7* |
|  | *sat2* | Phenicol resistance | 13052..13576 |  |
|  | *aadA1* | Aminoglycosides resistance | 13634..14422 |  |
